# Describing the application of statistical shape modelling to DXA images to quantify the shape of the proximal femur at ages 14 and 18 years in the Avon Longitudinal Study of Parents and Children

**DOI:** 10.12688/wellcomeopenres.15092.2

**Published:** 2019-08-27

**Authors:** Monika Frysz, Jenny S. Gregory, Richard M. Aspden, Lavinia Paternoster, Jonathan H. Tobias

**Affiliations:** 1 MRC Integrative Epidemiology Unit, University of Bristol, Bristol, UK; 2 Musculoskeletal Research Unit, Translational Health Sciences, University of Bristol, Bristol, UK; 3 Institute of Medical Science, University of Aberdeen, Aberdeen, UK

**Keywords:** ALSPAC, hip shape, joint shape, statistical shape modelling

## Abstract

Bones are complex objects with considerable variation in the shape and structure often attributed to anatomical, environmental or genetic differences. In addition, bone shape has been of interest in relation to its associations with disease processes. Hip shape is an important determinant of hip osteoarthritis and osteoporotic hip fracture; however, its quantification is difficult. While previous studies largely focused on individual geometrical indices of hip geometry such as neck-shaft angle or femoral neck width, statistical shape modelling offers the means to quantify the entire contour of the proximal femur, including lesser trochanter and acetabular eyebrow. We describe the derivation of independent modes of variation (hip shape mode scores) to characterise variation in hip shape from dual-energy X-ray absorptiometry (DXA) images in the Avon Longitudinal Study of Parents and Children (ALSPAC) offspring, using statistical shape modelling. ALSPAC is a rich source of phenotypic and genotypic data which provides a unique opportunity to investigate the environmental and genetic influences on hip shape in adolescence, as well as comparison with adult hip shape.

## Introduction

Bones are complex objects with each bone showing considerable variation in size and shape between individuals, which can be attributed to anatomical differences, environmental and genetic influences or be a consequence of a disease process. Traditionally these differences have been assessed by measuring lengths and angles, however it has been recognized that single geometrical measurements are often correlated with measures of body size as well as other geometrical indices ^1^. Statistical shape modelling (SSM) is a method which uses a set of landmark points to describe an outline of an object as opposed to a single geometrical measurement and can represent a combination of several different aspects of shape of that object (e.g. in case of proximal femur, concomitant variations in femoral neck (FN) and femoral head size and shape).

Musculoskeletal disorders are a significant cause of disability worldwide and the number of people affected is expected to increase given the ageing population, rise in obesity and increasingly sedentary lifestyles ^2^. Osteoarthritis (OA) and osteoporotic fractures are the most common age-related musculoskeletal diseases and are associated with significant healthcare burden. Previous studies suggest that hip shape is an important risk factor for both hip OA ^3, 4^ and osteoporotic hip fracture ^5^. Little is known, however, about its development in childhood and adolescence. Statistical shape modelling provides a means for capturing the global shape of the proximal femur; it uses principal components analysis to generate modes of variation (Hip Shape Modes (HSMs)) which describe each image in terms of standard deviations below or above the mean shape, after removing variation in size. One disadvantage of SSM in previous literature has been that models reflect the variation within the dataset they were trained on, making direct quantitative comparison between similar studies difficult. This can however be overcome by using a previously built model as a reference model for a subsequent dataset ^6, 7^.

The Avon Longitudinal Study of Parents and Children (ALSPAC) is a longitudinal birth cohort, which in the 1990s recruited pregnant women in South West England ^8^. ALSPAC is a rich source of data, including phenotypic and genetic data collected for the mothers, fathers and children. It is uniquely suited for examining variation in hip shape in earlier life, based on hip dual-energy X-ray absorptiometry (DXA) scans obtained when the children were, on average, 14 and 18 years old. This data note describes the methodology and data used to quantify the shape of the proximal femur in ALSPAC offspring at these time points. In order to allow direct comparability with other studies and between the time points, an adult reference statistical shape model (SSM) template (based on 19,379 images from 5 cohorts ^9^) was applied to these data.

These generated data (HSMs describing variation in hip shape) provide an opportunity to quantify variation in hip shape and be subsequently used in future analyses to examine sex differences in hip shape, and to explore associations with other factors, including genetic.

## Methods

### ALSPAC Data

ALSPAC is a longitudinal birth cohort which recruited a total of 14,541 pregnant women with expected delivery date between 1 ^st^ April 1991 and 31 ^st^ December 1992. Of these pregnancies, 69 have no known birth outcome, and of the remaining 14,472 pregnancies, 195 were twin, 3 were triplet and 1 was quadruplet accounting for 14,676 known foetuses. These pregnancies resulted in 14,062 live births, of which 13,988 children were alive at 1 year of age.

In addition to the initial enrolment that took place between 1991 and 1992, further recruitment took place when the children were, on average, 7 years old, and another from age 8 onwards to which eligible children and those not initially enrolled were also invited. This resulted in a total of 15,247 pregnancies enrolled. Since recruitment these children have been followed up at regular intervals; questionnaire and clinical assessment data have been collected. Moreover, additional data on siblings, mothers and their partners, have also been collected.

### Hip DXA scans

Hip DXA scans collected during two assessment clinics, Teen Focus (TF) 2 and TF 4, were used to quantify the shape of proximal femur. TF 2 was performed between January 2005 and September 2006. The target age for attendance was 13.5 years (mean age at attendance was 13.8 years, range 12.5–15.1 years). TF 4 clinic started in December 2008 and was completed by early to mid-2011. The target age for attendance was 17.5 years (mean age at attendance was 17.8 years, range 16.2–19.8 years).

Of 11,351 individuals invited to the TF 2 clinic, 6,147 attended and a total of 6,162 images were available to align in Shape software (please note that for quality purposes a number of individuals were re-invited and duplicate scans were performed), of which 4,468 were available for SSM. Of 10,101 individuals invited to the TF 4 clinic, 5,217 attended and 4,746 images were available to align in Shape, of which 4,413 were available for final modelling. For details regarding image exclusion please refer to Table 1.

**Table 1.** Avon Longitudinal Study of Parents and Children offspring hip shape data.

	Age 14	Age 18
Description	N	
Total number of images uploaded in shape	6,162	4,746
Excluded twins, sibs and re-invites	171	115
Excluded images without genetic or TF4 data	1,255 *	NA
Excluded images due to poor image quality	268	218
Total hips aligned	**4,468**	**4,413**
Of those, with genetic data	3,929	3,198
Of those, with data at both adolescent time points	3,188	

*Due to delay in image acquisition and given the time constrains, halfway through image alignment it was decided to restrict alignment of the remaining images to those who had both, genetic data and DXA image acquired at TF 4 clinic.

### Statistical shape model (SSM)

**Figure 1.**
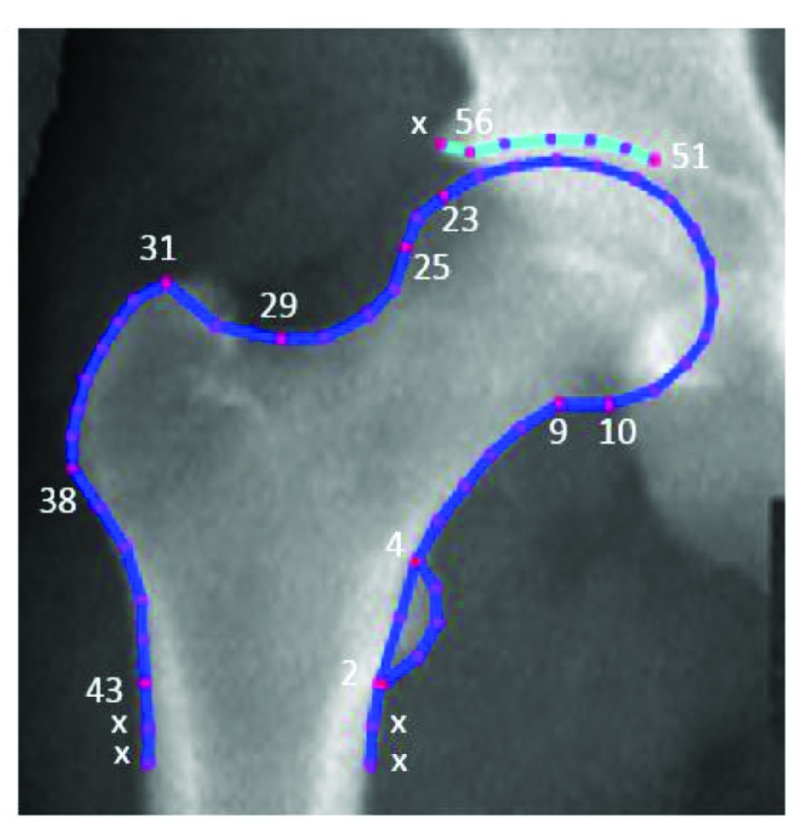
Outline of proximal femur shape and key landmark point positions used to derive 53-point SSM.

Raw hip DXA images were securely transferred to collaborators in Aberdeen for image processing and uploaded into Shape software (University of Aberdeen). Each image was marked up with a set of landmark points, which relate to points that are placed at easily identifiable anatomical features of an object (please refer to Figure 1, which shows the placement of landmark points, and Table 2, which describes the anatomical positions of each of the key landmark points (shown in red in Figure 1)).

**Table 2.** Description of the key landmark points shown in red in Figure 1.

Point number	Anatomical feature
**2**	Medial femoral shaft meets inferior lesser trochanter (often maps to point 46, depending on position)
**4**	Medial femoral shaft meets superior lesser trochanter
**9**	Change in curvature: lateral inferior curvature of femoral head at point where it meets femoral neck
**10**	Change in curvature: medial inferior curvature of the femoral head
**23**	Change in curvature: superior lateral femoral head curvature
**25**	Change in curvature: inferior lateral femoral head where meets the superior femoral neck
**29**	Inferior greater trochanter slope where it meets superior femoral neck
**31**	Medial superior greater trochanter
**38**	Inferior lateral greater trochanter
**43**	Lateral femoral shaft
**46**	Inferior lesser trochanter (often maps to point 2)
**51**	Acetabular eyebrow medial end (end of brightest line)
**56**	Acetabular eyebrow lateral end

Please note points 0, 1, 44, 45, and 57 [marked with x] were not included in the final model.

Following point placement, Procrustes analysis was used to estimate the mean shape. The aim of this step is, first, to remove any translational, rotational and scaling information and then align each image as closely as possible. Any effect of age and/or sex or other non-image variables is not accounted for at this stage. After completing the alignment, principal component analysis (PCA) was performed using the coordinates of each point to build the SSM, producing a set of orthogonal modes of variation known as principal components (referred to as hip shape modes (HSMs)). These modes together explain 100% of variance in the data set, with the first HSM accounting for the largest amount of variance and subsequent HSMs accounting for less variance. Each HSM has a mean of zero and unit standard deviation (SD), and each image and, consequently, each individual is assigned a set of values for each HSM which describes the number of SDs away from the mean shape.

### Applying external adult reference SSM template to adolescent data

One of the limitations of statistical shape modelling is the lack of comparability of HSMs with other datasets and studies, since each SSM is unique to that particular set of images. One way of overcoming this limitation is to apply a set of pre-defined HSMs, previously obtained from a reference population. An SSM template based on a reference set generated from a GWAS meta-analysis of hip shape from five cohorts (based on 19,379 images) ^9^, was applied to both adolescent datasets in order to directly compare hip shape between adolescent time points as well as with adult hip shape. See Table 3 for details regarding cohorts contributing to the adult reference SSM. Briefly, the reference model was built as described above and the eigenvectors were saved and used to calculate the mode scores for subsequent models (without adding the new image to the reference model or changing it in any way).

**Table 3.** Cohorts contributing to the adult reference statistical shape model.

Cohort	N	Gender	Mean age (SD) of participants
**ALSPAC mothers**	4,603	Females	47.9 (4.3)
**Framingham**	3,088	Males and females	63.3 (11.0)
**MrOS**	5,924	Males	74.0 (6.0)
**SOF**	1,715	Females	72.8 (4.6)
**Twins UK**	4,049	Males and females	52.5 (13.5)
**Total**	19,379		

ALSPAC, Avon Longitudinal Study of Parents and Children; MrOS, Osteoporotic fractures in men study; SOF, Study of Osteoporotic Fractures.

### Reproducibility of point placement

**Figure 2.**
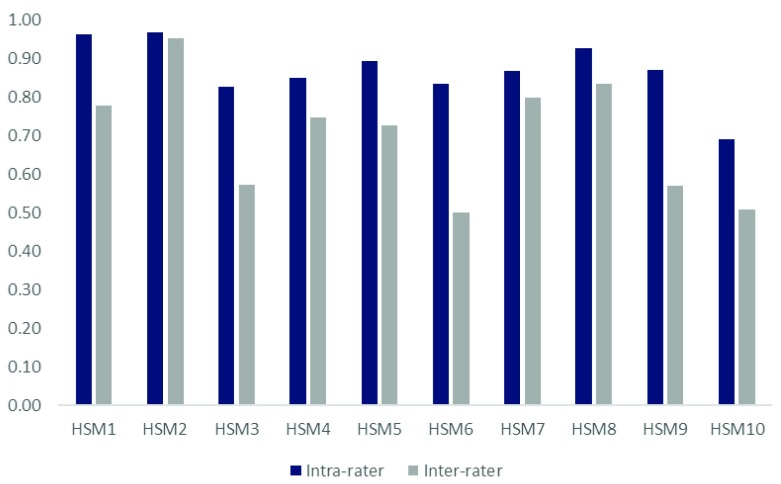
Intraclass Correlation Coefficients (ICCs) for the top ten HSMs.

A set of 100 images, collected during TF 4 clinic, were randomly selected and marked 2 months after completing the initial point placement in ALSPAC adolescents. The same set of images was also marked by a second marker. Intra- (within-) and inter-observer (between-observer) repeatability of manual point placement was measured as the difference in pixels between coordinates of 58 points. The intra- and inter-observer reliability assessed by mean point-to-point repeatability was 1.22 and 1.78 pixels, respectively. Considering that the average size of hip DXA image in pixels was 250 × 180, these errors are small and a cut off median point-to point difference of less than or equal to 3 was previously considered as accurate ^10^. In addition, average Intraclass Correlation Coefficients (ICCs) for the top ten HSMs were calculated. Figure 2 shows the intra- and inter-observer agreement values for each of the modes. The mean ICC values were 0.87 for intra- and 0.70 for inter-observer agreement. Whilst all ICC values for intra-observer agreement were above or equal to 0.70, inter-rater scores for modes 3, 6, 9 and 10 were below 0.70. Whilst the initial model was based on a 58-point model, this was subsequently modified to a 53-point model due to high variability in points placed at the acetabular overhang and medial and lateral femoral shaft, in both adolescent and adult SSM templates.

### Dataset

**Figure 3.**
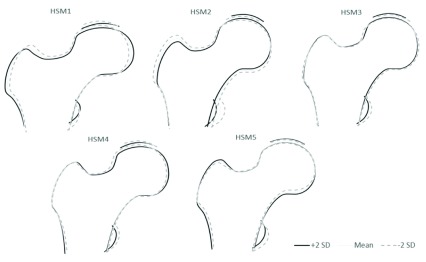
Variation in hip shape described by modes 1-5, based on adult reference SSM.

**Figure 4.**
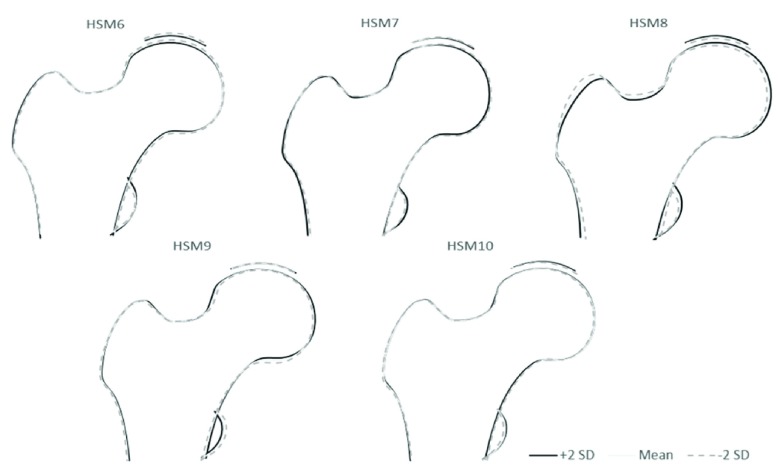
Variation in hip shape described by modes 6-10, based on adult reference SSM.

The first ten HSM scores generated using external adult reference SSM for adolescent data collected at ages 14 and 18 years, are available in the ALSPAC resource. A total of 4,468 individuals had hip shape data generated at age 14 (2,140 were male, 2,328 were female) and total of 4,413 had data available at age 18 (1,939 were males, 2,474 were female). Please refer to Table 4 for descriptive statistics of the final sample for ALSPAC adolescents. Similarly to previously published literature ^10– 12^ the first 10 modes, which together explained 85% of variance, were selected (higher modes >10 can often be regarded as noise as each represents less than 1.5% of the variance). Figure 3 and Figure 4 provide graphical representation and Table 5 provides summary of the features described by each HSM. Compared to mean = 0 and SD = 1 when using the data as its own reference, when using the adult reference SSM (based on adult data with age ranging from 48 to 74 years), means for the first ten HSMs ranged from -1.14 to 2.26 at age 14 and from -1.5 to 2.42 at age 18, whereas SDs ranged from 0.42 to 0.97 at age 14 and from 0.41 to 0.91 at age 18 ( Table 6).

**Table 4.** Characteristics of ALSPAC participants.

		Age 14			Age 18		
Variable	Group	N	Mean (SD)	Range	N	Mean (SD)	Range
Age at clinic (years)	Combined	4,467	13.8 (0.2)	(12.5;15.2)	4,413	17.8 (0.4)	(16.3;19.8)
	Males	2,140	13.8 (0.2)	(12.5;15.1)	1,939	17.8 (0.4)	(16.4;19.8)
	Females	2,327	13.8 (0.2)	(12.6;15.2)	2,474	17.8 (0.4)	(16.3;19.8)
Height (cm)	Combined	4,467	163.4 (7.7)	(131.8;193.0)	4,413	171.1 (9.2)	(143.6;208.0)
	Males	2,139	165.1 (8.7)	(131.8;193.0)	1,939	178.7 (6.6)	(153.3;208.0)
	Females	2,327	162.0 (6.2)	(134.9;183.9)	2,474	165.2 (6.2)	(143.6;196.1)
Weight (kg)	Combined	4,464	54.6 (11.0)	(26.6;125.4)	4,413	67.1 (13.6)	(39.4;144.0)
	Males	2,139	54.8 (11.6)	(26.6;106.4)	1,939	72.5 (13.2)	(45.7;144.0)
	Females	2,324	54.4 (10.4)	(30.2;125.4)	2,474	62.9 (12.4)	(39.4;139.2)
BMI	Combined	4,464	20.4 (3.4)	(13.6;44.2)	4,413	22.9 (4.1)	(14.7;48.2)
	Males	2,139	20.0 (3.3)	(13.8;35.8)	1,939	22.7 (3.8)	(15.2;46.1)
	Females	2,324	20.7 (3.5)	(13.6;44.2)	2,474	23.0 (4.3)	(14.7;48.2)

Abbreviations: BMI (body mass index)

**Table 5.** Variation described by the top ten modes based on adult reference SSM. Please refer to Figure 3 and Figure 4 for graphical representation of each mode.

HSM (% of variation)	Key features described by each mode: +2 SDs (solid line) -2 SDs (dashed line)
1 (42%)	Positive scores (solid line) - Loss of femoral head curvature - Narrower FN Negative scores (dashed line) - Wider FN Smaller NSA
2 (13%)	Positive scores (solid line) - Narrower FN and femoral shaft - Smaller greater trochanter - Smaller femoral head (inferior aspect proximal to lesser trochanter) Negative scores (dashed line) - Wider FN - Larger greater and lesser trochanters
3 (8.5%)	Positive scores (solid line) - Smaller lesser trochanter - Narrower FN Negative scores (dashed line) - Wider FN - Larger lesser trochanter
4 (6.1%)	Positive scores (solid line) - Larger femoral head (medial aspect) - Narrower FN - Smaller lesser trochanter Negative scores (dashed line) - Cam-type deformity - Wider FN
5 (4.1%)	Positive scores (solid line) - Larger femoral head (inferior aspect proximal to lesser trochanter) - Larger greater trochanter - Wider FN Negative scores (dashed line) - Smaller femoral head (inferior aspect proximal to lesser trochanter) - Narrower FN - Larger lesser trochanter
6 (3.4%)	Positive scores (solid line) - Narrower FN Negative scores (dashed line) - Wider FN
7 (2.6%)	Positive scores (solid line) - Wider femoral shaft Negative scores (dashed line) - Narrower femoral shaft - Smaller lesser trochanter
8 (2.5%)	Positive scores (solid line) - Larger femoral head - Narrower FN - Smaller greater trochanter Negative scores (dashed line) - Smaller femoral head - Wider FN - Larger greater trochanter
9 (1.8%)	Positive scores (solid line) - Smaller femoral head (inferior aspect proximal to lesser trochanter) - Smaller lesser trochanter Negative scores (dashed line) - Larger femoral head (inferior aspect proximal to lesser trochanter) - Larger lesser trochanter
10 (1.5%)	Positive scores (solid line) - Larger lesser trochanter Negative scores (dashed line) - Smaller lesser trochanter

FN, femoral neck; NSA, neck-shaft angle.

**Table 6.** Mean HSM scores for the top ten HSMs based on ALSPAC adolescent and mothers’ images, after applying adult reference SSM (compared with mean=0 and SD=1 when data from each time point included as its own reference).

	Age 14	Age 18	Mothers
HSM	Mean (SD)	Mean (SD)	Mean (SD)
1	2.26 (0.42)	2.42 (0.41)	1.45 (0.53)
2	0.57 (0.76)	0.23 (0.85)	-0.01 (0.90)
3	-0.19 (0.68)	0.10 (0.66)	-0.31 (0.92)
4	0.87 (0.68)	0.36 (0.73)	0.32 (0.77)
5	-1.14 (0.79)	-1.50 (0.84)	-0.35 (0.94)
6	0.27 (0.68)	0.27 (0.86)	-0.01 (1.00)
7	-0.25 (0.63)	0.02 (0.70)	-0.14 (0.87)
8	0.39 (0.97)	0.02 (0.91)	0.06 (0.95)
9	0.22 (0.76)	-0.21 (0.91)	0.34 (0.95)
10	-1.09 (0.59)	-1.04 (0.77)	0.11 (0.92)

When the adult reference SSM was applied to ALSPAC mothers’ images, means for HSMs 2–9 were close to 0 (ranging from -0.35 to 0.34) and SDs were close to 1 (ranging from 0.8 to 1), whereas mean and SD HSM1 score were 1.45 and 0.5, respectively.

The differences in means and SDs could be due to sex and/or age differences (i.e. mothers were on average 48 years old, therefore more closely resembling the ages of cohorts included in the reference model as opposed to ALSPAC offspring). The deviation away from the mean was particularly noted for HSM1, which is likely to reflect scanner differences between ALSPAC and other cohorts in the adult reference set. Different pixel spacing in the Lunar Prodigy scanner (used to acquire DXA scans in ALSPAC) relative to other scanners alters the aspect ratio (ratio between image height and width), and therefore HSM1 reflects these differences. Likewise, the smaller standard deviation is likely to reflect the narrower range generated when only one scanner is used.

Whilst direct comparison of the modes across the time points is an added advantage of applying an external reference SSM, one of the potential issues that may arise is that previously independent HSMs might no longer be independent of each other. In order to quantify the extent of the potential loss of independence, after applying SSM based on the combined adult reference model to adolescent data Matrix Spectral Decomposition was performed using the matSpD tool to compute the number of independent modes. The top ten HSMs based on adult reference SSM at both time points were first correlated ( Table 7 and Table 8) and tested for independent number of variables (HSMs) using matSpD. As expected, the results showed that the top ten HSMs were essentially independent, as reflected by matSpD score of 9.6, indicating 4% loss of independence.

**Table 7.** Correlation matrix for the top ten HSM scores at age 14 to assess the number of independent variables using matrix Spectral Decomposition (matSpD) which showed strong evidence for nearly all variables (9.6) to be independent.

	HSM1	HSM2	HSM3	HSM4	HSM5	HSM6	HSM7	HSM8	HSM9	HSM10
**HSM1**	1	0.1853	0.0371	0.0375	0.4698	-0.198	0.1578	-0.272	-0.2019	-0.1227
**HSM2**	0.1853	1	0.4216	0.131	0.3872	0.0883	0.054	-0.118	0.3098	-0.1471
**HSM3**	0.0371	0.4216	1	0.2081	0.1451	-0.0381	0.1772	0.144	0.2597	-0.1564
**HSM4**	0.0375	0.131	0.2081	1	0.0924	-0.1778	0.248	0.1602	0.2208	-0.2277
**HSM5**	0.4698	0.3872	0.1451	0.0924	1	-0.2271	0.0095	-0.0648	0.3164	-0.0647
**HSM6**	-0.198	0.0883	-0.0381	-0.1778	-0.2271	1	-0.0972	0.1324	-0.2759	-0.0347
**HSM7**	0.1578	0.054	0.1772	0.248	0.0095	-0.0972	1	-0.3302	0.2572	0.0019
**HSM8**	-0.272	-0.118	0.144	0.1602	-0.0648	0.1324	-0.3302	1	-0.191	0.0862
**HSM9**	-0.2019	0.3098	0.2597	0.2208	0.3164	-0.2759	0.2572	-0.191	1	-0.1126
**HSM10**	-0.1227	-0.1471	-0.1564	-0.2277	-0.0647	-0.0347	0.0019	0.0862	-0.1126	1

**Table 8.** Correlation matrix for the top ten HSM scores at age 18 to assess the number of independent variables using matrix Spectral Decomposition (matSpD) which showed strong evidence for nearly all variables (9.6) to be independent.

	HSM1	HSM2	HSM3	HSM4	HSM5	HSM6	HSM7	HSM8	HSM9	HSM10
**HSM1**	1	0.141	0.2264	-0.0047	0.4621	-0.2515	0.0537	-0.1779	-0.1618	-0.0226
**HSM2**	0.141	1	0.3793	0.1983	0.4458	-0.1167	0.1083	-0.1985	0.3159	-0.0712
**HSM3**	0.2264	0.3793	1	0.4535	0.1827	-0.1872	0.3169	-0.0169	0.0756	-0.1303
**HSM4**	-0.0047	0.1983	0.4535	1	0.0864	-0.1524	0.1849	0.204	0.1695	-0.2213
**HSM5**	0.4621	0.4458	0.1827	0.0864	1	-0.3191	0.0347	-0.1862	0.4001	-0.0575
**HSM6**	-0.2515	-0.1167	-0.1872	-0.1524	-0.3191	1	-0.1257	0.1897	-0.3383	-0.0189
**HSM7**	0.0537	0.1083	0.3169	0.1849	0.0347	-0.1257	1	-0.1477	0.2756	0.1138
**HSM8**	-0.1779	-0.1985	-0.0169	0.204	-0.1862	0.1897	-0.1477	1	-0.1628	0.1194
**HSM9**	-0.1618	0.3159	0.0756	0.1695	0.4001	-0.3383	0.2756	-0.1628	1	-0.0967
**HSM10**	-0.0226	-0.0712	-0.1303	-0.2213	-0.0575	-0.0189	0.1138	0.1194	-0.0967	1

SSM methodology offers a powerful approach to study subtle changes in hip morphology and it has been successfully applied to study variation in hip shape associated with the incidence ^13, 14^ and progression of OA ^15^, as well as associations with hip fracture ^16^ in adult cohorts. A major drawback of the methodology has previously been that as each model is data-driven, the HSMs generated are unique to the sample used, thus preventing direct cross-comparison with other studies. One of the key strengths of hip shape data presented here is the application of an adult reference SSM to hip DXA images at ages 14 and 18 years, which allows direct comparisons of associations with HSMs between these time points and comparison of findings with results in adults. For example using the results from the largest to date meta-analysis of DXA derived hip shape ^9^, we were able to replicate these analyses in adolescents and directly compare the relationships between genetic loci associated with hip shape in adults with those in adolescents ^17^. Furthermore, future analyses examining associations between hip shape and OA-case status, applying the same SSM template which was used for the purpose of this data note will enable future studies in adolescents to focus on those aspects of hip morphology more strongly related to pathology in later life.

## Ethical approval and consent

Ethical approval for the study was obtained from the ALSPAC Ethics and Law Committee and the Local Research Ethics Committees, full details of the approvals obtained are available from the study website ( http://www.bristol.ac.uk/alspac/researchers/research-ethics/).

Written informed consent was obtained from parents, and children were invited to give consent where appropriate. Study members have the right to withdraw their consent for elements of the study or from the study entirely at any time.

## Data availability

ALSPAC data access is through a system of managed open access. The steps below highlight how to apply for access to the data included in this data note and all other ALSPAC data. The dataset generated in this data note has been deposited within the ALSPAC data resource and is linked to ALSPAC project number B1274. Please quote this number to request required variables which have been described in this dataset (HSMs generated at ages 14 and 18 years).

1. Please read the ALSPAC access policy (PDF, 627kB) which describes the process of accessing the data and samples in detail, and outlines the costs associated with doing so.
2. You may also find it useful to browse our fully searchable research proposals database, which lists all research projects that have been approved since April 2011.
3. Please submit your research proposal for consideration by the ALSPAC Executive Committee using the online process. You will receive a response within 10 working days to advise you whether your proposal has been approved.

If you have any questions about accessing data, please email alspac-data@bristol.ac.uk.

The ALSPAC data management plan describes in detail the policy regarding data sharing, which is through a system of managed open access.
