# Supplementary material for: Describing the application of statistical shape modelling to DXA images to quantify the shape of the proximal femur at ages 14 and 18 years in the Avon Longitudinal Study of Parents and Children
Source: Wellcome Open Res. 2019 Aug 27;4:24. Originally published 2019 Feb 6. [Version 2] doi: 10.12688/wellcomeopenres.15092.2 (PMC6733378; doi:10.12688/wellcomeopenres.15092.2)
Supplement: Peer review history

# Peer review: Reviewer response for version 2

**DOI:** 10.21956/wellcomeopenres.16860.r36316

**Published:** 2019-09-03

**Authors:** Carme Rissech

**Affiliations:** 1 Unit of Human Anatomy and Embriology, Department of Medical Sciences, Faculty of Medicine and Health Sciences, Rovira i Virgily University, Reus, Spain

The authors have addressed my comments and suggestions appropriately in the revised version. The new version is indexable.

I confirm that I have read this submission and believe that I have an appropriate level of expertise to confirm that it is of an acceptable scientific standard.

# Peer review: Reviewer response for version 2

**DOI:** 10.21956/wellcomeopenres.16860.r36315

**Published:** 2019-08-27

**Authors:** Martha C. Castaño-Betancourt

**Affiliations:** 1 Laboratory of Genetics Epidemiology, Jundiaí (FMJ), Jundiaí Medical School, Jundiaí, Brazil

I consider that the authors provided the changes that were pointed by the reviewers. I do not have further comments.

I confirm that I have read this submission and believe that I have an appropriate level of expertise to confirm that it is of an acceptable scientific standard.

# Peer review: Reviewer response for version 1

**DOI:** 10.21956/wellcomeopenres.16465.r35146

**Published:** 2019-04-15

**Authors:** Martha C. Castaño-Betancourt

**Affiliations:** 1 Laboratory of Genetics Epidemiology, Jundiaí (FMJ), Jundiaí Medical School, Jundiaí, Brazil

The article presented by Frysz et al, examined variation in hip shape using scans (DXA) obtained in children on average, 14 and 18 years old, applying statistical shape modelling.

 Points to be addressed:

1. "Applying external adult reference SSM template to adolescent data": The comparison was done using similar modes between cohorts? or the first ten modes? What is exactly the reference model? Please describe how or what is the reference model. 
2. "The intra- and inter-observer reliability assessed by mean point-to-point repeat ability was 1.22 and 1.78 pixels, respectively". I would like to see a known estimate of intra-class correlation because in pixels it does not tell much to the readers of the article.  The question is: the correlation within and between reader was good? What are the estimates?
3. "The differences in means and SDs could be due to sex and/or age differences" it means that the HSMs were not adjusted for age and gender? Please clarify in methods.
4. In methods, how many females, range of age, BMI...do you have information on those variables? If you have it please add it.
5. In my opinion HSM1 could be use as reference for the other modes. It represents the gross percentage of variation. If this mode (HSM1) what really reflect is scanner differences, when you correct in your equation for type of scanner the difference would disappear. Can you confirm this?
6. "The results showed close to 10 (9.6) independent variables for both time points" Please clearly describe in the document whether the 10 modes were independent or not (or which of them were not) and what was the real loss of independence (in % or number that readers understand). 
7. In table 6 and 7 what is represented in the axis X and Y to make the correlation?

I confirm that I have read this submission and believe that I have an appropriate level of expertise to confirm that it is of an acceptable scientific standard, however I have significant reservations, as outlined above.

# Response

**Published:** 2019-08-20

**Authors:** Monika Frysz

**Affiliations:** University of Bristol, UK

*Points to be addressed:*

*"Applying external adult reference SSM template to adolescent data": The comparison was done using similar modes between cohorts? or the first ten modes? What is exactly the reference model? Please describe how or what is the reference model.*

 We thank the referee for their helpful comments.

 The reference model is a numerical description of a set of images using, in this case, a previous model based on N=19,379 images. By default, PCA produces a number of HSMs explaining 100% of variation in the dataset. However, as in most previous publications, the top few modes explain the majority of the variance in the dataset and ten were selected for analysis. The reference model is locked and HSMs describing each image from the adolescent cohort data were calculated in terms of this model. Again, we selected the top ten for analysis in order to be able to compare them with the previous study. The number of modes selected for analysis, however, has no bearing on the results of the reference model (so had we chosen 9 or 90 modes; the values would be unaffected).

 We have added additional text to explain this point at suitable points in the manuscript and provided references to other studies that have used reference models in this way.

*"The intra- and inter-observer reliability assessed by mean point-to-point repeat ability was 1.22 and 1.78 pixels, respectively". I would like to see a known estimate of intra-class correlation because in pixels it does not tell much to the readers of the article.  The question is: the correlation within and between reader was good? What are the estimates?*

 We have calculated intra-class correlation coefficients as suggested (description and a figure showing the results have been added to the manuscript) and updated the sentence relating to pixel results to add clarity.

*"The differences in means and SDs could be due to sex and/or age differences" it means that the HSMs were not adjusted for age and gender? Please clarify in methods.*

 We have now clarified in methods that derived HSMs were not adjusted for age and gender.

*In methods, how many females, range of age, BMI...do you have information on those variables? If you have it please add it.*

 We have now added a table with descriptive statistics to the dataset section.

*In my opinion HSM1 could be use as reference for the other modes. It represents the gross percentage of variation. If this mode (HSM1) what really reflect is scanner differences, when you correct in your equation for type of scanner the difference would disappear. Can you confirm this?*

 SSM just provides equations for describing shape based on a set of points and does not have the facility for inputting other variables such as type of scanner, so it is not possible to formally correct HSM1 results for the type of scanner.

 HSM1, in the reference cohort, was found to be skewed due to different scanners used in the various cohorts in that study. The link between scanner-type and HSM1 does not have any direct bearing on the results for this study when examined alone however should be borne in mind if comparisons are being made with other studies using this reference model. Adjustments were made to reduce the differences between scanners in the previous study but, as highlighted, this is not relevant to this study as only a single scanner was used, so we have removed the last sentence, of the second paragraph in the dataset section, for clarity.

*"The results showed close to 10 (9.6) independent variables for both time points" Please clearly describe in the document whether the 10 modes were independent or not (or which of them were not) and what was the real loss of independence (in % or number that readers understand).*

 This has now been clarified and the following added:

 “As expected, the results showed that the top ten HSMs were essentially independent, as reflected by matSpD score of 9.6 indicating 4% loss of independence.”

*In table 6 and 7 what is represented in the axis X and Y to make the correlation?*

 Both tables represent mean HSM scores for each time point. X and Y values are the same. Of the top 10 HSMs, each HSM was correlated with each other in order to assess the independence of the modes.

# Peer review: Reviewer response for version 1

**DOI:** 10.21956/wellcomeopenres.16465.r34986

**Published:** 2019-03-08

**Authors:** Carme Rissech

**Affiliations:** 1 Unit of Human Anatomy and Embriology, Department of Medical Sciences, Faculty of Medicine and Health Sciences, Rovira i Virgily University, Reus, Spain

From the title, it seems that the objective of the paper is to quantify the shape of the human proximal femur at ages 14 and 18 years by using the DXA scans from the Avon Longitudinal Study of Parents and Children. However, at the end of the introduction section, in the objectives, the authors say: “This data note describes the methodology and data used to quantify the shape of the proximal femur in ALSPAC offspring at these time points” (14 and 18 years of age). This lack of alignment between the title and objectives confuses the reader. In addition, the Introduction section starts talking about osteoarthritis and osteoporothic fractures, which confuses the reader even more. If the goal of this paper is to describe the methodology and data that in other studies the authors will use, the authors should focus on that. Therefore, the authors should clearly define their objectives and construct the title of the paper and the Introduction section according these objectives. Both of them (Title and Introduction) should lead the reader to the final objectives of the paper.  I think that in addition to describing the methodology used to analyse the proximal shape of the femur by statistical shape modelling and the landmarks used, the authors should also explain how they will use all of this in their analyses. I wonder why they do not construct different models for boys and girls. It is well known that female and male femur each follow divergent growth trajectories which are clearly marked from 12 years of age onward (Pujol et al., 2016 ^1^). How are they going to use these ten 10 PCs on future papers?  How will the application of the external adult reference statistical shape model template to adolescents and mothers aid comparability with other studies and ages? What results do they think can obtain from the application of these data in their future analyses? I think that all of this should be better explained and discussed in the paper.

 Other comments:

- What is the difference between Geometric Morphometrics and Statistical shape modelling? This and the advantages to use Statistical shape modelling in front of Geometric Morphometrics should be explained in the Introduction section.
- What do key landmarks mean in Statistical shape modelling?
- In Material and Methods section, when the authors describe the final chosen sample of the Avon Longitudinal Study of Parents and Children used for their study, they should indicate the final number of boys and girls to be analysed.

I confirm that I have read this submission and believe that I have an appropriate level of expertise to confirm that it is of an acceptable scientific standard, however I have significant reservations, as outlined above.

## References

[1] : Ontogeny of the male femur: Geometric morphometric analysis applied to a contemporary Spanish population. *Am J Phys Anthropol*.2016;159(1) : 10.1002/ajpa.22846 146-63 10.1002/ajpa.22846 26331786

# Response

**Published:** 2019-08-20

**Authors:** Monika Frysz

**Affiliations:** University of Bristol, UK

*From the title, it seems that the objective of the paper is to quantify the shape of the human proximal femur at ages 14 and 18 years by using the DXA scans from the Avon Longitudinal Study of Parents and Children. However, at the end of the introduction section, in the objectives, the authors say: “This data note describes the methodology and data used to quantify the shape of the proximal femur in ALSPAC offspring at these time points” (14 and 18 years of age). This lack of alignment between the title and objectives confuses the reader.*

 We thank the referee for their helpful comments. The title has now been changed to:

 Describing the application of statistical shape modelling to DXA images to quantify the shape of the proximal femur at ages 14 and 18 years in the Avon Longitudinal Study of Parents and Children

*In addition, the Introduction section starts talking about osteoarthritis and osteoporothic fractures, which confuses the reader even more. If the goal of this paper is to describe the methodology and data that in other studies the authors will use, the authors should focus on that. Therefore, the authors should clearly define their objectives and construct the title of the paper and the Introduction section according these objectives. Both of them (Title and Introduction) should lead the reader to the final objectives of the paper.*

 We have now updated the introduction and abstract in line with this comment.

*I think that in addition to describing the methodology used to analyse the proximal shape of the femur by statistical shape modelling and the landmarks used, the authors should also explain how they will use all of this in their analyses.*

 In order to clarify the focus of this paper, we added the following to the introduction:

 “These generated data (hip shape modes (HSMs) describing variation in hip shape) provide an opportunity to quantify variation in hip shape and be subsequently used in future analyses to examine sex differences in hip shape, and to explore associations with other factors, including genetic.”

*I wonder why they do not construct different models for boys and girls. It is well known that female and male femur each follow divergent growth trajectories which are clearly marked from 12 years of age onward (Pujol et al., 2016 1).*

 We built mixed-gender model which allows direct comparison of each HSM between the sexes. In addition, mixed-gender model increases the sample size and allows for a richer model containing additional shape information.

*How are they going to use these ten 10 PCs on future papers?*

 These 10 PCs can be used as both, individual exposures and individual outcomes in future analyses to explore what risk factors and later outcomes are associated with hip shape Please refer to last paragraph of the introduction section where we suggested how these can be utilised in future studies.  

*How will the application of the external adult reference statistical shape model template to adolescents and mothers aid comparability with other studies and ages? What results do they think can obtain from the application of these data in their future analyses? I think that all of this should be better explained and discussed in the paper.*

 We have now added further details in the last paragraph of the dataset section.

*Other comments:*

*What is the difference between Geometric Morphometrics and Statistical shape modelling? This and the advantages to use Statistical shape modelling in front of Geometric Morphometrics should be explained in the Introduction section.*

 There is no material difference between these methodologies. The primary difference lies in how the results are displayed (direct variation in shape rather than warping of an underlying grid). In musculoskeletal research the SSM approach and terminology has been used in preference to GM so we prefer to continue to use this to avoid confusion.

*What do key landmarks mean in Statistical shape modelling?*

 Key landmark points in this data note relate to points that are placed at easily identifiable anatomical features of an object – this sentence has now been added to ‘Statistical shape model (SSM)’ section of the data note.

*In Material and Methods section, when the authors describe the final chosen sample of the Avon Longitudinal Study of Parents and Children used for their study, they should indicate the final number of boys and girls to be analysed.*

 This has now been added to the dataset section.
